# Wastewater Surveillance to Estimate and Characterize Hepatitis E Virus Circulation

**DOI:** 10.1007/s12560-025-09644-4

**Published:** 2025-05-21

**Authors:** C. Dimeglio, O. Schlosser, S. Laperche, C. De Smet, S. Demmou, J. Latour, N. Jeanne, M. Tribout, N. Bleuez, J. Figoni, F. Abravanel, S. Lhomme, J. Izopet

**Affiliations:** 1 Laboratoire de Virologie, Centre National de Référence Virus de l’Hépatite E, CHU Toulouse-Purpan, 31059 Toulouse, France; 2 INFINITY, INSERM U1291, CNRS 5051, Université Paul Sabatier Toulouse III, Toulouse, France; 3 Suez, Centre International de Recherche sur l’Eau et l’Environnement (CIRSEE), 78230 Le Pecq, France; 4 Etablissement Français du Sang, 93218 Saint-Denis, France; 5 Etablissement Français du Sang Occitanie, 31059 Toulouse, France; 6 Etablissement Français du Sang Nord-Pas-de-Calais, 59012 Lille, France; 7 Santé Publique France, 94410 Saint-Maurice, France

**Keywords:** HEV RNA, Wastewater, Blood donors, Long-read SMRT, Modeling

## Abstract

Hepatitis E virus (HEV) is a cause of enterically transmitted hepatitis around the world. Because of the high frequency of asymptomatic infections, the magnitude of HEV infection is underestimated. Wastewater monitoring could be useful to improve our knowledge on HEV epidemiology. In this study, we analyzed the capacity of wastewater surveillance to give an insight into the circulation and the diversity of HEV in two French cities. HEV RNA was detected and quantified by digital PCR in 115 untreated composite wastewater samples collected weekly at the inlet of wastewater treatment plants (WWTPs), 58 at Toulouse WWTP and 57 at Dunkerque WWTP. Plasma HEV RNA in blood donors was detected by a commercial assay (Roche Cobas) over the same period in the same area. HEV diversity was analyzed using long-read single-molecule real-time sequencing (Pacific Biosciences). HEV RNA was detected in 88% and 95% wastewater samples collected at Toulouse (Occitanie region, Southern France) and Dunkerque (Hauts-de-France region, Northern France) WWTPs, respectively. HEV RNA concentration ranged between 4.1 and 5.7 log copies/L and was almost similar between the two sites. A long *orf2* fragment of HEV genome (1030 nucleotides) was obtained and sequenced in 45% and 70% of positive HEV RNA wastewater samples collected at Toulouse site and Dunkerque site, respectively. Out of 31 strains identified in Toulouse wastewater, 24 were HEV-3c (77%), 6 were HEV-3f (19%), and 1 was HEV-3h (3%). Out of 55 strains identified in Dunkerque, 30 were HEV-3c (55%) and 25 were HEV-3f (45%). All HEV RNA-positive samples from blood donors that could be genotyped during the study period contained HEV-3. Subtype distribution in 51 blood donors living in Toulouse did not differ from that in Toulouse wastewater. The HEV-3 subtype distribution in 51 Hauts-de-France region blood donors and in Dunkerque wastewater were different, but the predominant subtype was the same (HEV-3c). Lastly, we explored the link between the measurement of viral loads in wastewater and the extent of infection in the served population. Although a good correlation between the peaks of positive HEV RNA estimated in wastewater samples and that observed in blood donors was observed with a lag of + 3 weeks for Toulouse, the correlation was weaker for Dunkerque. Wastewater surveillance system applied locally could be very useful for assessing the HEV infection status of a population.

## Introduction

Hepatitis E virus (HEV) is a major cause of hepatitis worldwide leading to frequent asymptomatic infections but also severe illness including acute liver failure in pregnant women and people with underlying chronic liver disease (Kamar et al., 2017). HEV is also the cause of chronic hepatitis progressing rapidly toward cirrhosis in immunocompromised individuals (Kamar et al., 2017). In addition, HEV infection is associated with extrahepatic manifestations including renal and neurological disorders (Dalton et al., 2016; Kamar et al., 2017). HEV (Family *Hepeviridae*, species *Paslahepevirus balayani* according to International Committee on Taxonomy of Viruses 2022) has 8 genotypes (HEV-1-8), with HEV-1-4 being the most widespread. HEV-1 and 2, predominant in Asia and Africa, exclusively infect humans and are mainly transmitted by drinking water contaminated by feces. In contrast, HEV-3 predominant in Europe and America and HEV-4 present mainly in Asia are zoonotic viruses circulating in reservoir animals (pigs, wild boars, deers, and rabbits). They infect humans by direct contact or by ingestion of raw or undercooked meat and meat products prepared from infected animals. HEV-3 is endemic in most European countries, including France (Izopet et al., 2019). Studies in blood donors using standardized assays suggest that the level of endemicity varies from one country to another (Domanovic et al., 2017), and from one area to another within the same country (Mansuy et al., 2016; Spada et al., 2022).

Despite accumulation of knowledge on HEV during the past 20 years and international recommendations for diagnostic testing (European Association for the Study of the Liver. Electronic address 2018), HEV infection remains underdiagnosed and its impact on public health is underestimated. Therefore, effective measures to mitigate transmission cannot be taken. Although wastewater-based surveillance (WBS) has been used for monitoring enteric viruses including poliovirus since the late 1930s, the COVID-19 pandemic has revealed the transformative potential of this approach for studying infectious diseases and their impact on public health (Parkins et al., 2024; Singer et al., 2023). Several studies have shown that HEV can be detected in wastewater all over the world (Casares-Jimenez et al., 2024; Clemente-Casares et al., 2009; Giron-Guzman et al., 2024; Pina et al., 1998; Rau et al., 2024; Salemane et al., 2024; Treagus et al., 2023; Vaidya et al., 2002; Veneri et al., 2024). However, quantitative data and their interpretation are still limited because symptomatic HEV infections are relatively rare in the general population, at least in high income countries, and asymptomatic infections are not identified (Abravanel et al., 2025; Kamar et al., 2017).

The aim of this study was to analyze the capacity of wastewater surveillance to estimate HEV circulation (incidence rate, genotypes) in people living in two French cities, Toulouse (Occitanie region, Southern France) and Dunkerque (Hauts-de-France region, Northern France). We determined the level of agreement between HEV genomic diversity in wastewater and the one assessed in infected individuals. HEV diversity was analyzed using long-read single-molecule real-time sequencing allowing both accurate genotyping and haplotyping. Data from wastewater surveillance and systematic screening of HEV RNA in blood donations in the same area were compared.

## Material and Methods

### Wastewater Samples

A total of 115 untreated wastewater samples were weekly collected between January 2023 and March 2024: 58 samples at the southern inlet to Toulouse wastewater treatment plant (WWTP) receiving the excreta of approximately 323 000 inhabitants (40% of Toulouse Métropole population) and 57 samples at the inlet to Dunkerque WWTP receiving the excreta of 88 266 inhabitants (46% of the Urban Community of Dunkerque population). WWTPs are hereinafter referred to as Toulouse WWTP and Dunkerque WWTP. Twenty-four-hour flow-proportional composite samples were collected weekly using automatic sampling devices, preserved, transported at 5°, and delivered to the IAGE (Ingenierie et Analyse en Génétique Environnementale, Montpellier, France) laboratory for analysis within 24 h. Data of flow rate at the WWTP inlet were available for each sampling day. The sewer system is combined at Dunkerque WWTP, i.e., both wastewater and stormwater flow through the same pipes, whereas the sewer system at Toulouse WWTP is mainly separate. Toulouse and Dunkerque WWTP do not receive effluents from pig farms, slaughterhouses, meat-processing plants or agricultural run-off.

### HEV Screening of Blood Donations

Systematic HEV genomic screening of blood donations performed by the EFS (French Blood Establishment) based on the Roche Cobas HEV assay (limit of detection 95%: 18 IU/ml) was used to estimate HEV incidence in Toulouse and Dunkerque areas. From March 2023 to December 2023, 76,178 samples in the Nord department (Hauts-de-France) and 40,587 samples taken in Haute-Garonne (Occitanie) were analyzed. Incidence results are given per week. HEV-RNA-positive donations indicated an overall incidence of 1.85/1000 donations in Occitanie administrative region and 0.68/1000 donations in Hauts-de-France administrative region (Laperche et al., 2024). Viral load was measured by RT-PCR using the AltoStar HEV1.5 (Altona Germany) method (limit of detection 95%: 10 IU/ml). HEV-RNA-positive donations were characterized by single-molecule real-time sequencing at the National Reference Center for HEV (Toulouse University Hospital).

### Viral Concentration and RNA Extraction

HEV was concentrated by ultrafiltration of wastewater samples using anisotropic membrane by IAGE as previously reported (Viveros et al., 2022). Briefly, 30 ml of each wastewater sample were homogenized by gentle shaking before 15 ml were processed to recover total RNA. Nucleic acid extraction from wastewater was performed after ultrafiltration using magnetic particles (Indical Bioscience, Leipzig, Germany) following the manufacturer’s instructions. Pepper Mild Mottle Virus (PMMoV) was used as internal control of wastewater RNA extraction. The extracted RNA was eluted in a final volume of 100 µl of elution buffer.

### HEV RNA Detection and Quantification

Digital PCR was performed by IAGE following the manufacturer’s instructions (Qiagen, Germany) using the QIAcuity Eight 5-plex Platform system, the QIA cuity One-step Viral RT-PCR kit, and QIAcuity 26K 24-well Nanoplates (Viveros et al., 2022). Primers used target the *orf3/orf2* overlapping region of HEV genome (Frias et al., 2021). For the targeted sequence of PMMoV, the probe and primers were previously described (Kitajima et al., 2018). Data were analyzed using the QIAcuity Software Suite 2.5.0.1 (Qiagen, Germany) and expressed as copies/L of wastewater sample.

### HEV RNA Characterization by Long-Read Single-Molecule Real-Time Sequencing

A 1039 nucleotide fragment within the *orf2* region of the HEV genome was sequenced using PacBio technology on a Sequel IIe instrument (Pacific Biosciences), as previously reported (Abravanel et al., 2025). Briefly, HEV RNA was reverse transcribed using the Superscript IV Vilo Master Mix (Thermo Fisher) enzyme. The RT-PCR program was set at 25 °C for 10 min, 60 °C for 8 min, and 85 °C for 10 min. The *orf2* region was amplified using Platinum SuperFi II (Invitrogen). The PCR cycle was run at 98 °C for 2 min followed by 40 cycles at 98 °C for 15 s, 65 °C for 20 s, and 72 °C for 1 min using the following primers: 5′-GTAAAACGACGGCCAGTGTGTGTGTATTCTCAGCCCTT CGC-3′ 5′-CAGGAAACAGCTATGACGCCGAAATYAATTCTGTCGG-3′. A barcoding PCR using the Kapa HiFi Hotstart ReadyMix (Roche) and barcoded primers with the sequence of the M13 floating tail (TIB molBiol, Berlin, Germany) allowed sample identification with the following program: 95 °C for 5 min, 32 cycles at 98 °C for 20 s, 65 °C for 15 s, and 72 °C for 1 min.

The presence and quality of a long ORF2 fragment at the expected size was determined by capillary electrophoresis on a Fragment Analyzer Systems (Agilent technologies, Santa Clara, California). PCR barcoding products were quantified on a Light Cycler 480 Real-Time PCR instrument (Roche) with equimolar pooling. PCR products were then sequenced on a Sequel® IIe instrument in accordance with the manufacturer’s instructions. HiFi reads were generated on the instrument using the PacBio circular consensus sequencing (CCS) mode.

Bioinformatics analysis was carried out with a Snakemake workflow. The demultiplexing of HiFi reads with lima (v.2.6.0, https://lima.how/) was performed on reads with a quality threshold > Q30. The minimum coverage depth was 200 reads to analyze the results. Haplotypes were created using pbAA (PacBio tool, v.1.0.2 https://github.com/PacificBiosciences/pbAA), and only those with a read count exceeding 2% of the total number of reads were used for further analyses. Absence of inter-sample contamination was verified using a homemade script, identifying strictly identical haplotypes between samples on a subregion discarding extremities. HEV genotype and subtype were determined using reference sequences proposed by Smith et al. (2020). The sequences were aligned with MAFFT (v7.505) and a bootstrap tree (ultra-fast bootstrap—1000 replicates) was inferred using the maximum likelihood method (GTR + F + R10 evolution model) with IQ-TREE (v2.0.3).

The presence of strain mixtures in wastewater was confirmed by duplicate experiment and phylogenetic analysis.

### Statistical Analysis

Two sources of data from Toulouse (Haute-Garonne, Occitanie) and Dunkerque (Nord, Hauts-de-France) were used:

i. The rate of HEV RNA positivity obtained from blood donations screening (Y).
ii. HEV RNA load per 1000 (genome copies/day/1000 persons) in wastewater samples denoted ${L}_{\text{RNA}}$

${L}_{\text{RNA}}$ was calculated as follows: ${L}_{\text{RNA}}$ = *C*_ww_ × *Q*_d_ × 1000/*P*,

where *C*_ww_ is the concentration on HEV RNA measured in wastewater sample (in gc/L), *Q*_d_ is the daily wastewater inlet flow rate at the time of sampling (in L/day), and *P* is the served population (in number of inhabitants).

The mass of excreted stool ($\text{MS}$) can be estimated at 128 g/day (Rose et al., 2015), the density of stool (${d}_{S}) \text{at} 1.06 \text{g}/\text{mL}$ (Brown et al., 1996). Based on previous studies (Goel et al., 2020; Sottil et al. 2024; Takahashi et al. 2007), median HEV RNA concentration in stool samples, can be estimated at 8.3 × 10^7^ copies/ml (${C}_{\text{RNA}}$), ranging from 10^2^ to 10^8^ copies/ml according to the time of sampling after the peak of ALT activity.

The percentage of infected individuals could be

$$X={L}_{\text{RNA}}/(\text{MS}\times \frac{1}{{d}_{\text{S}}}\times 1000\times {\text{C}}_{\text{RNA}} ).$$

To obtain an estimate of the frequency of positive HEV RNA in the general population from the HEV flux data obtained in the wastewaters, we were looking for a simple linear relationship between *Y* and *X* as follows:

$$Y=\beta X+\varepsilon.$$

We could correct the amplitude discrepancies between the frequencies of positive HEV RNA from blood donors (*Y*) and from wastewater samples (X) as follows:

$$I=\alpha \times {L}_{\text{RNA}}/(\text{MS}\times \frac{1}{{d}_{S}}\times 1000\times {\text{C}}_{\text{RNA}} ),$$

where

$$\alpha =\frac{1}{\beta }.$$

Data were analyzed using Stata version 14® (StataCorp LP, College Station, TX, USA). Differences in metrics of HEV RNA were evaluated using univariate analyses. Chi-squared and Fisher’s exact tests, Student’s *t* test, or Mann–Whitney *U* test were used when appropriate. Statistical significance was set at *p* < 0.05.

## Results

### Detection of HEV RNA in Wastewater and Blood Samples

Out of 58 samples collected at Toulouse WWTP from January 2023 to March 2024, 51 (88%) were HEV-RNA positive by digital PCR. Out of 57 samples collected at Dunkerque WWTP from January 2023 to March 2024, 54 (95%) were HEV-RNA positive. PMMoV was detected in all the analyzed samples at high level (4.6 × 10^7^ to 8 × 10^8^ genome copies/L) with no difference between Toulouse and Dunkerque. The detection rates of HEV RNA in wastewater from both sites were similar and no seasonality was identified (*p* > 0.05).

The frequency of positive HEV RNA in blood donations in Toulouse from March 2023 to December 2023 was 87 out of 40,587 giving a prevalence rate of 2.1/1000 (95% CI 1.8/1000–3.1/1000). This prevalence rate was stable during the study period in agreement with wastewater data. The frequency of positive HEV RNA in blood donations in Dunkerque from March 2023 to December 2023 was 65 out of 76,178 donations giving a prevalence rate of 0.8/1000 (95% CI 0.3/1000–1.5/1000) that was lower compared to the prevalence rate in Toulouse (*p* < 0.05). However, this rate was sufficient for inducing a very high frequency of HEV RNA detection in wastewater.

### Quantification of HEV RNA

**Fig. 1:**
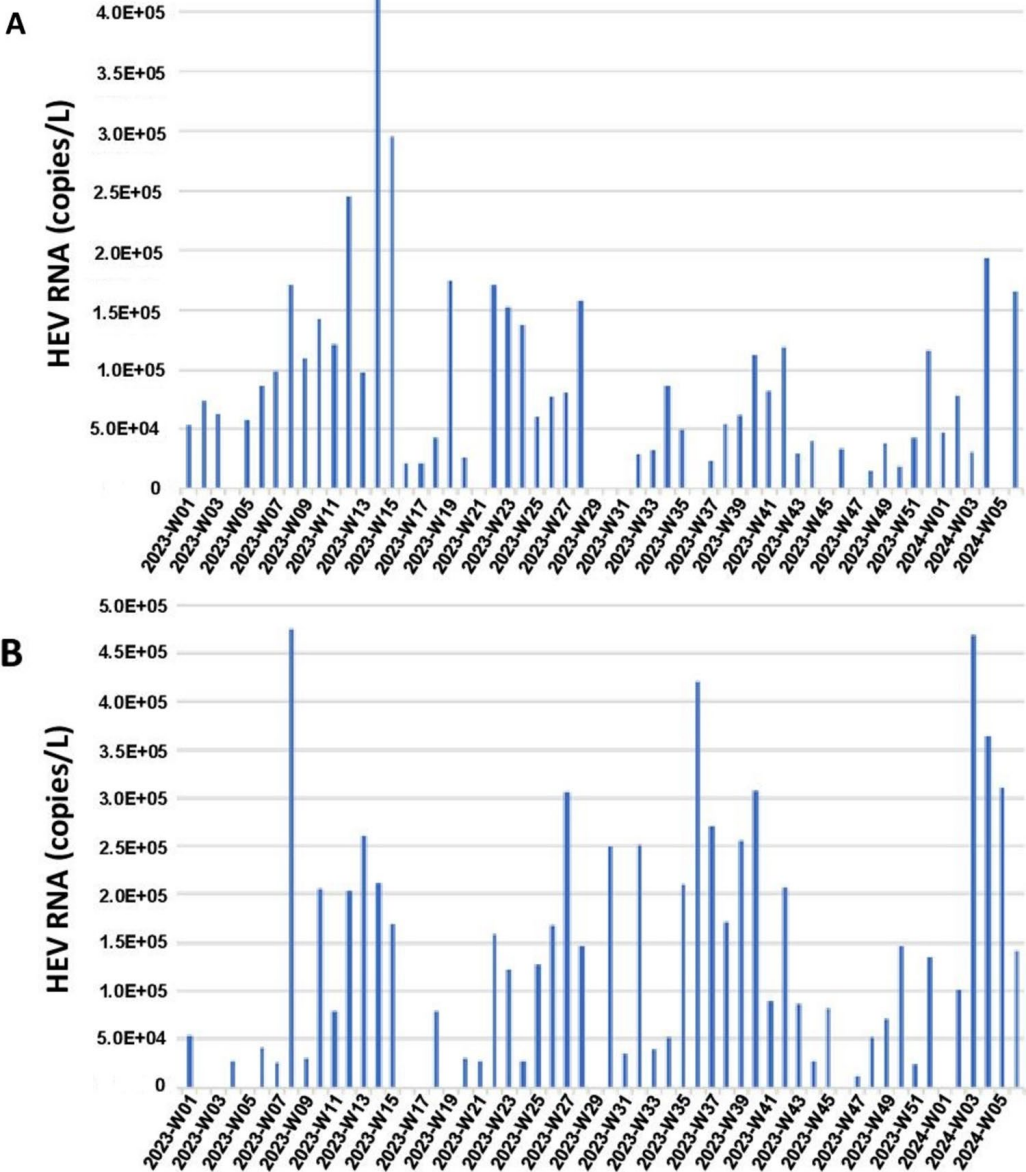
Time series of the HEV RNA concentration in wastewater—**A** Toulouse WWTP. **B** Dunkerque WWTP

**Fig. 2:**
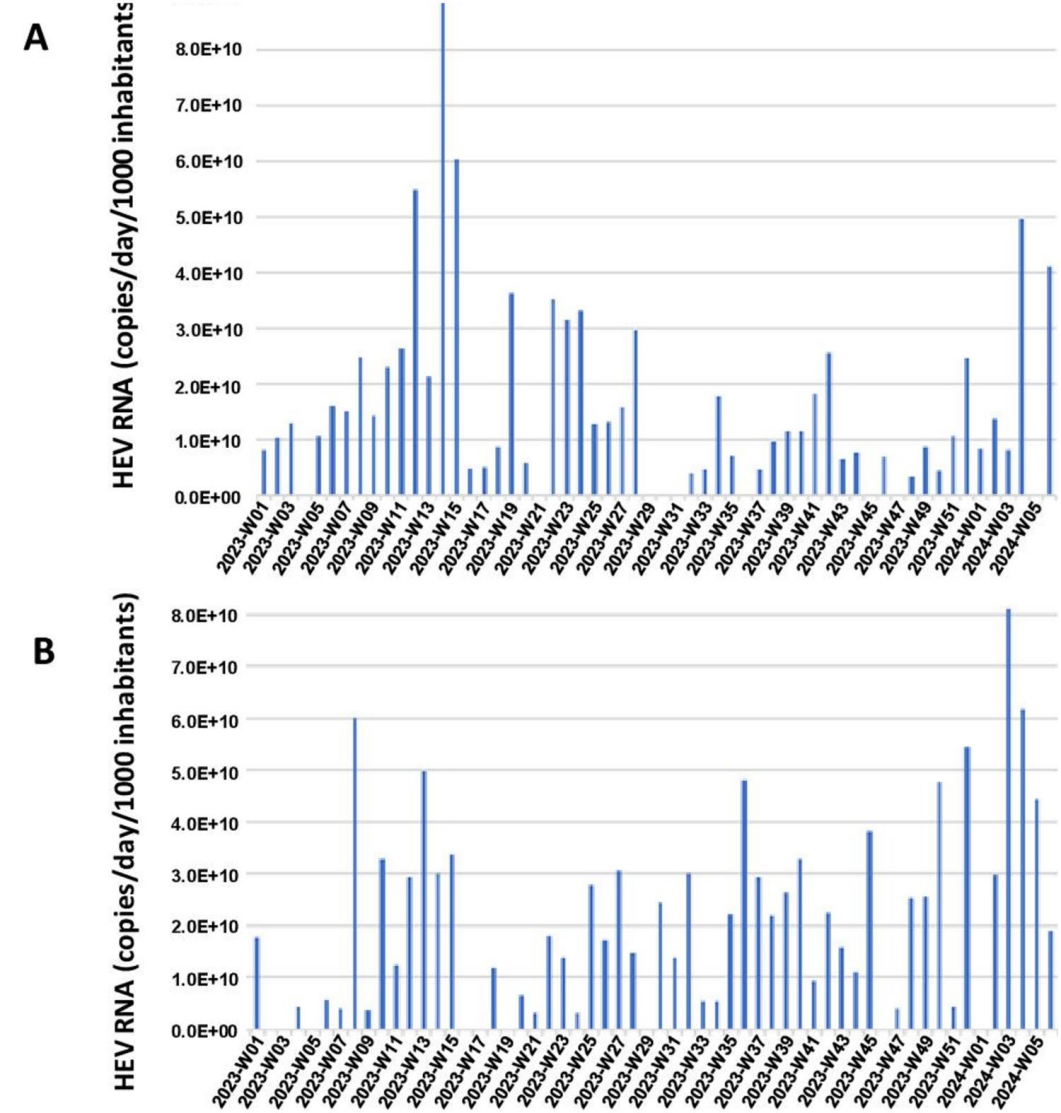
Time series of the HEV RNA load in wastewater: **A** Toulouse WWTP. **B** Dunkerque WWTP

Median HEV RNA concentration in wastewater samples from Toulouse WWTP was 4.9 log copies/L, ranging from 4.2 log copies/L to 5.6 log copies/L (Fig. 1A). Median HEV RNA concentration in wastewater samples from Dunkerque WWTP was 5.1 log copies/L ranging from 4.1 log copies/L to 5.7 log copies/L (Fig. 1B). After normalization of the concentration on the daily flow rate and the size of the population served, there was no significant difference in HEV RNA load per day/1000 inhabitants in wastewater samples from Toulouse and Dunkerque (Mann–Whitney test, *p* = 0.144) (Fig. 2).

### Characterization of HEV RNA

**Fig. 3:**
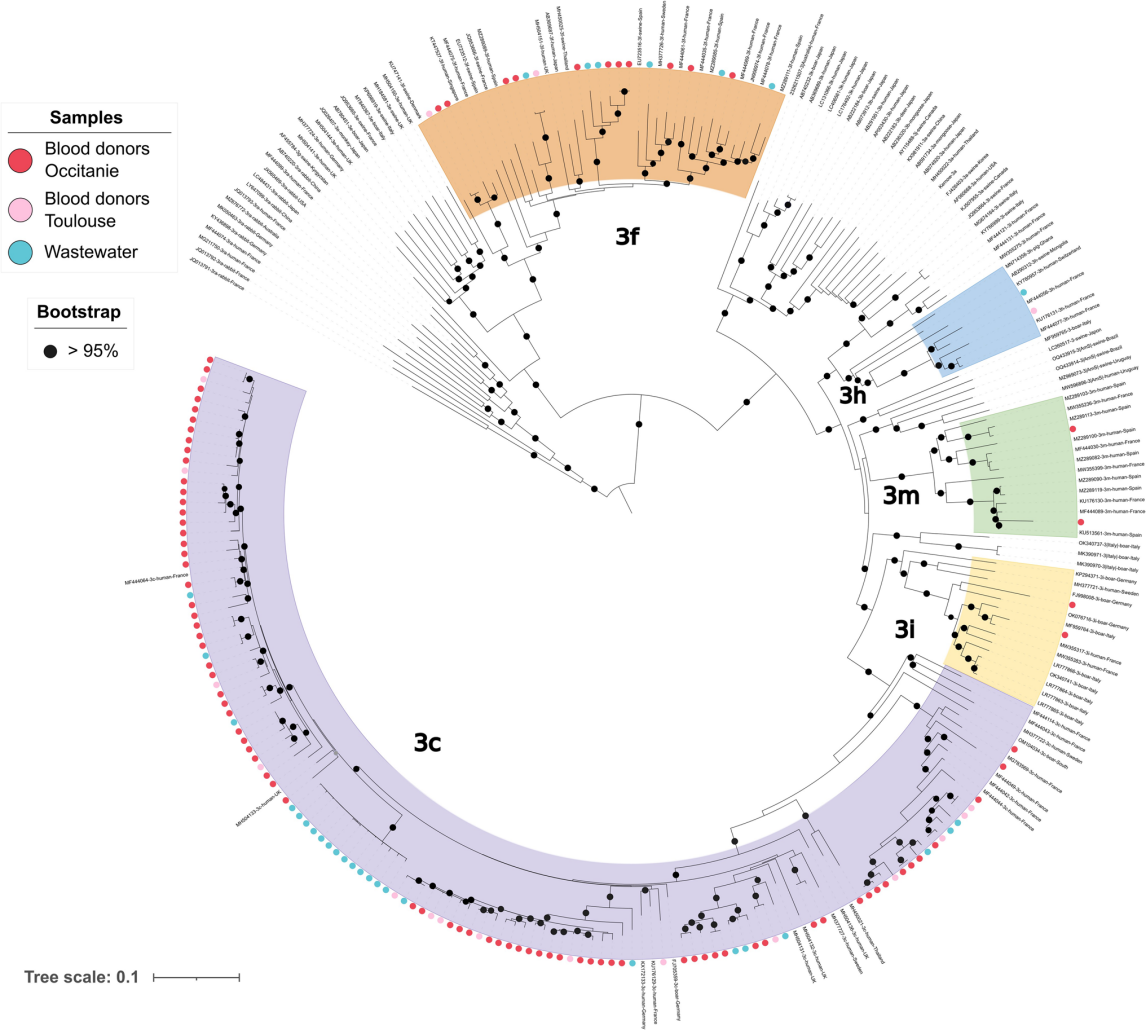
Phylogenetic analysis of HEV sequences of strains detected in wastewater samples from Toulouse WWTP (blue circles). HEV sequences of strains detected from March 2023 to March 2024 in blood donors living in Toulouse (pink circles) and in Occitanie outside of Toulouse (red circles)

Out of 51 HEV-RNA positive wastewater samples collected at Toulouse site, a long *orf2* fragment was obtained and sequenced in 23 samples (45%). Phylogenetic analysis showed a pattern indicating the presence of a single strain in 17 samples (74%) and the presence of a mixture (26%) of 2 and 3 strains in 4 and 2 samples, respectively. A total of 31 strains were identified (Fig. 3). All the strains belonged to genotype 3 and among them, 24 were subtyped 3c (78%), 6 were subtyped 3f (19%), and one was subtyped 3 h (3%).

**Fig. 4:**
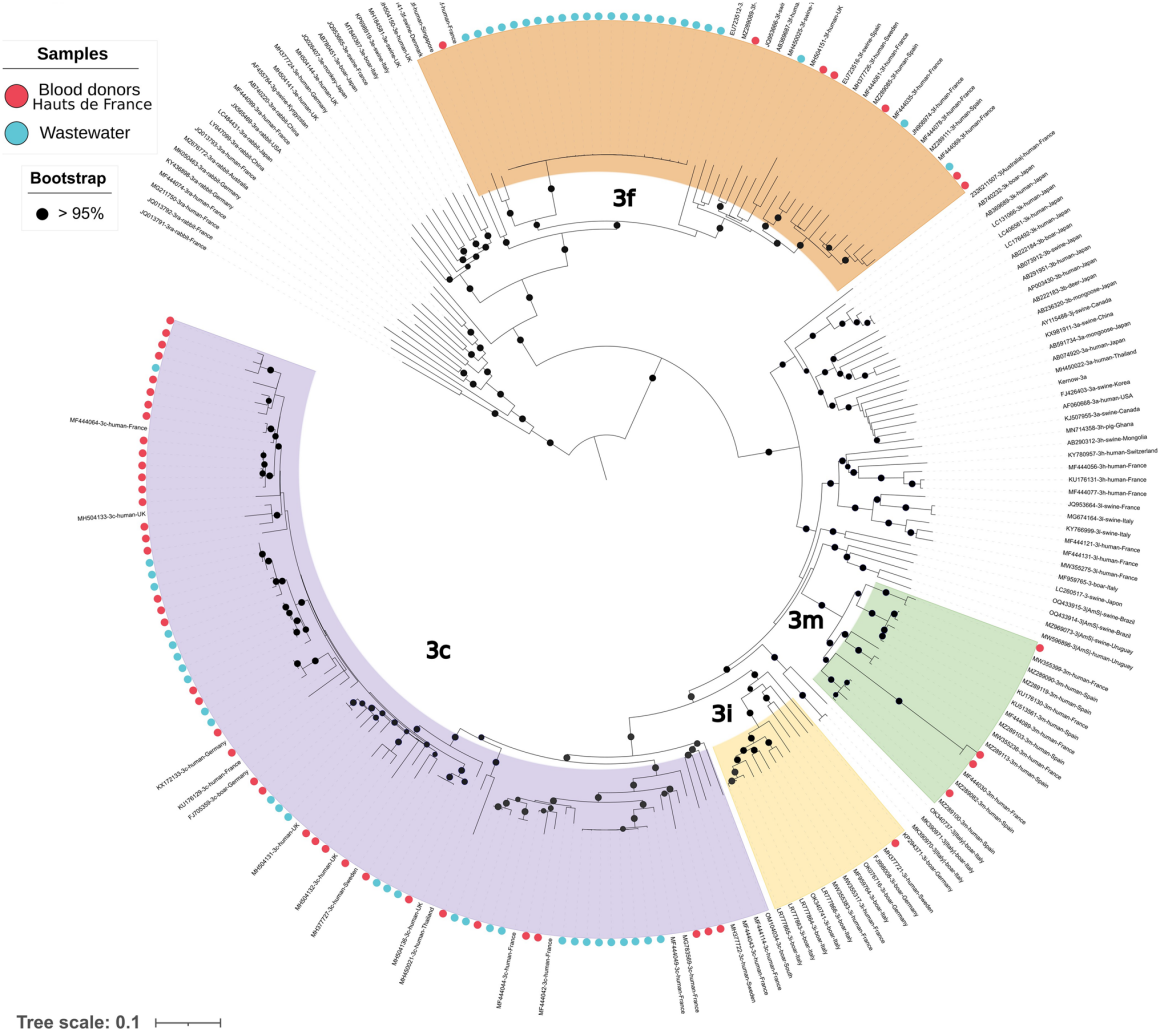
Phylogenetic analysis of HEV sequences of strains detected in wastewater samples from Dunkerque WWTP (blue circles). HEV sequences of strains detected from March 2023 to March 2024 in blood donors living in Hauts-de-France (red circles)

Out of 54 HEV RNA-positive wastewater samples collected at Dunkerque site, a long *orf2* fragment was obtained and sequenced in 38 samples (71%). Phylogenetic analysis showed a pattern indicating the presence of a single strain in 24 samples (63%) and the presence of a mixture (37%) of 2 and 3 strains in 11 and 3 samples, respectively. A total of 55 strains were identified (Fig. 4). All were genotype 3 and out of these, 30 were subtyped 3c (55%) and 25 were subtyped 3f (45%). Distribution of the subtypes was significantly different between samples taken at Toulouse WWTP and those taken at Dunkerque WWTP (Fischer’s exact test, *p* = 0.018).

In blood donations, all the samples that could be genotyped during the study period contained HEV-3. The distribution of HEV-3 subtypes in 108 donations in Occitanie was 3c (83%), 3f (12%), 3 h (1%), and other subtypes (4%). Moreover, the distribution of HEV-3 subtypes in 17 blood donors living in Toulouse was 3c (83%), 3f (11%), and 3 h (5%). Distribution of subtypes did not significantly differ between blood donations in Occitanie region and those in Toulouse city (Fischer’s exact test, *p* = 0.628).

**Fig. 5:**
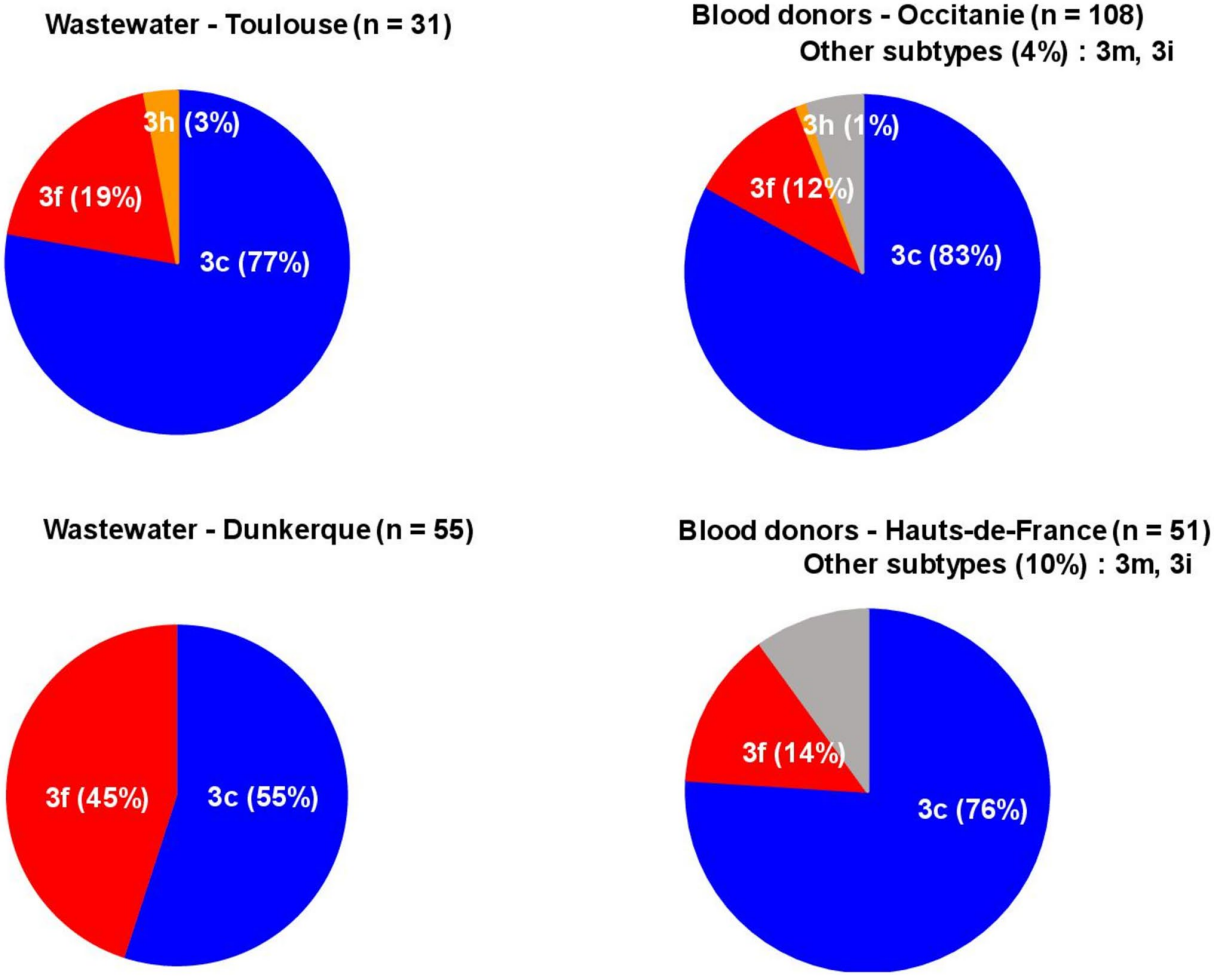
Distribution of HEV-3 subtypes in wastewater (Toulouse and Dunkerque) and in blood donors from the two regions (Occitanie and Hauts-de-France)

There was no significant difference in the distribution of subtypes between wastewater samples from Toulouse WWTP and blood donors both in the Toulouse (*p* = 1.0) and in the Occitanie regions (*p* = 0.595) (Fig. 5).

The distribution of the HEV-3 subtypes in Hauts-de-France was 3c (76%), 3f (14%), and other subtypes (10%), but the distribution of HEV-3 subtypes in blood donors living in Dunkerque could not be determined due to the limited number of genotyped strains. The distribution of the HEV-3 subtypes in Hauts-de-France contrasted with the distribution of HEV-3 subtypes in wastewater samples from Dunkerque site (*p* < 0.01, Chi-square test) (Fig. 5).

### Modeling HEV Circulation from Blood Donors Screening and Wastewater Sampling

**Fig. 6:**
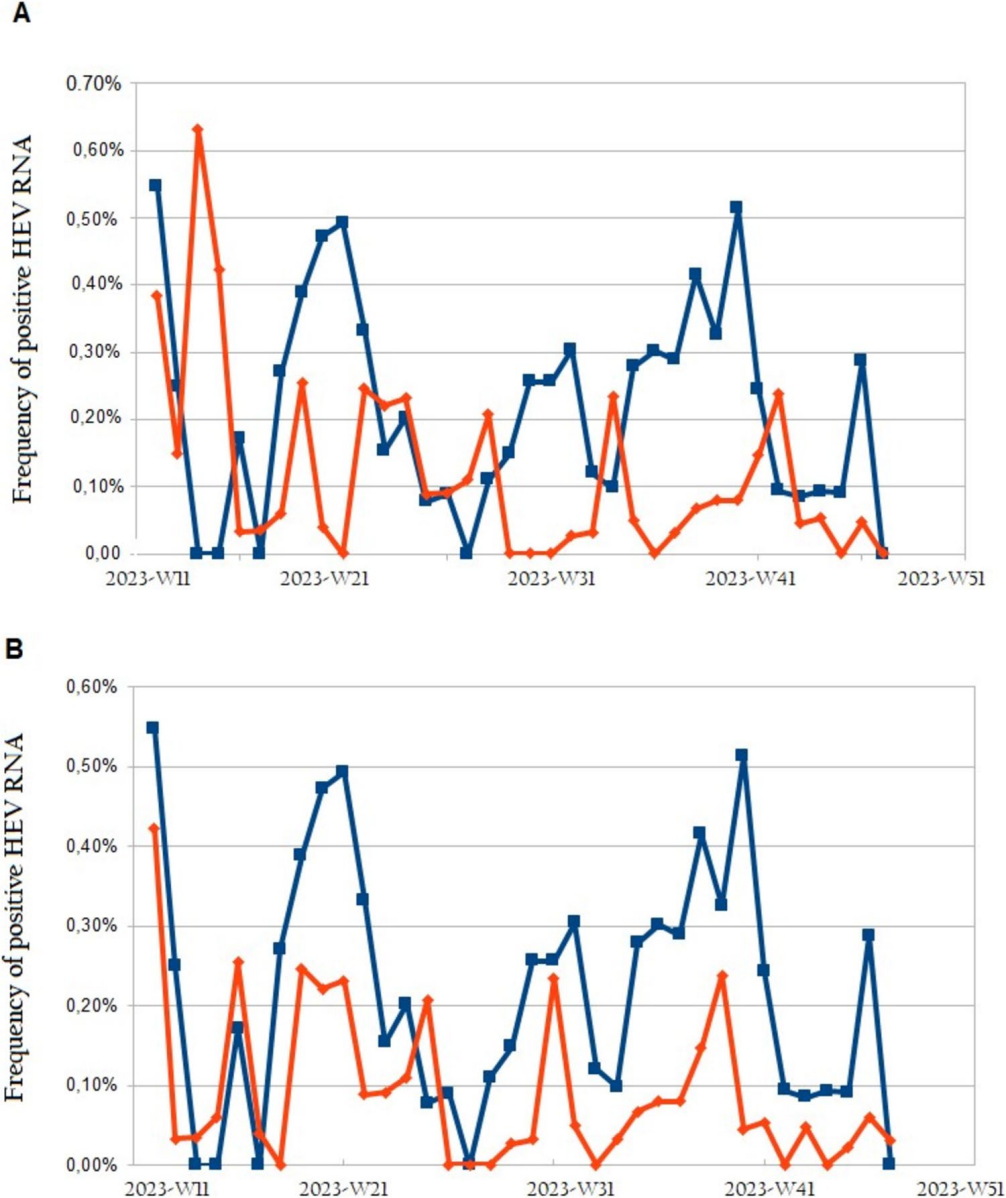
Frequency of positive HEV RNA in blood donors (blue curve) and estimated from the wastewater sampling (red curve) in Toulouse without lag in time (**A**) and with a 3-week lag (**B**)

**Fig. 7:**
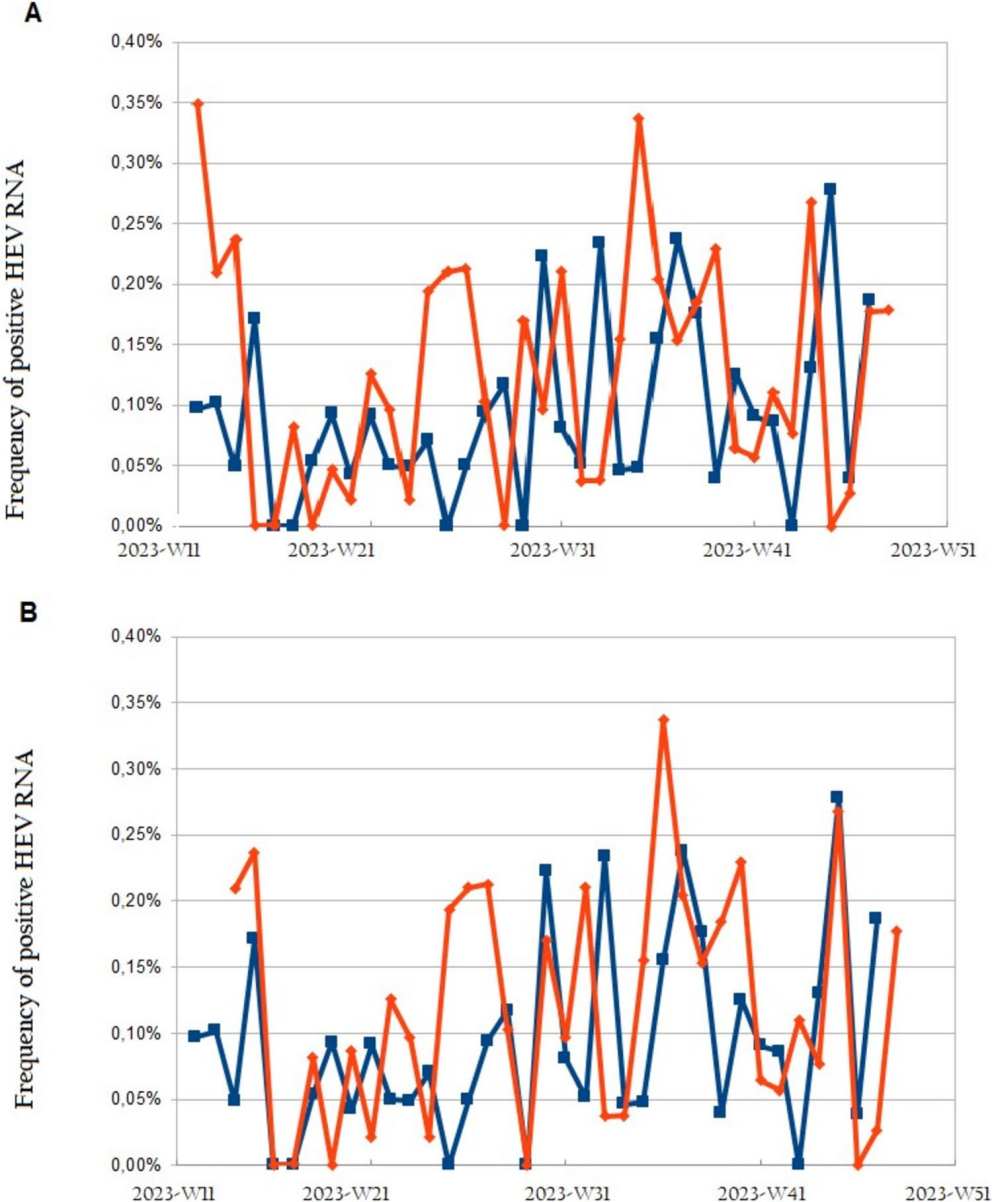
Frequency of positive HEV RNA in blood donors (blue curve) and estimated from the wastewater sampling (red curve) in Dunkerque without lag in time (**A**) and with a -1-week lag (**B**)

To demonstrate the potential interest of wastewater surveillance to analyze the occurrence of HEV infections, we estimated the rate of HEV RNA positivity in the population each week from the model using HEV RNA load in wastewater per 1000 inhabitants. At the same time, we reported the HEV RNA positivity rate observed each week in blood donations in Toulouse (Fig. 6) and Dunkerque (Fig. 7).

We observed a temporal shift of the peaks in HEV RNA positivity in blood donations and from wastewater data processing (Figs. 6A, 7A). Sometimes the peaks in wastewater samples occurred mainly after the peaks in blood donations (Toulouse, Fig. 6A) or sometimes the HEV RNA peaks in wastewater occurred before those in blood donations (Dunkerque, Fig. 7A). However, the shift of the peaks between wastewater and blood was not constant, even within the same city.

Regarding Toulouse, we observed on average a 3-week lag between the peaks in HEV RNA positivity from wastewater data processing and in blood donors (Fig. 6A and 6B). The coefficient of the linear regression linking data from blood donations and wastewater sampling data was *β* = 0.92 (*p* < 0.01) and the R-square of the model was 0.45.

In the case of Dunkerque, peaks in HEV RNA positivity from wastewater data processing preceded by one week those observed in blood donations (Fig. 7A and 7B). The coefficient of the linear regression linking data from blood and wastewater was *β* = 0.65 (*p* < 0.05) and the R-square of the model was 0.26.

## Discussion

HEV detection in sewage was first reported more than 30 years ago (Jothikumar et al., 1993). Subsequently, HEV was detected in water environments both in developing countries and in industrialized countries (Takuissu et al., 2022), but correlations with the frequency of infections in the population were never documented. In our study, we analyzed the rate of HEV RNA positivity, HEV RNA concentration and HEV diversity in wastewater samples and blood donations over the same period in two French cities, Toulouse and Dunkerque. To our knowledge, this study is the first one that reports HEV RNA data from both wastewater and blood donors in the same areas.

HEV RNA was detected in most wastewater samples. Our results are in line with the data reported in a study performed in Edinburgh, Scotland, UK, where 14/15 (93%) untreated sewage samples were HEV-RNA positive (Smith et al., 2016). In a German study, the detection rate was 84–100% (Beyer et al., 2020). A French study also reported that 10/11 (91%) samples from Nancy wastewater treatment plant were positive for *Paslahepevirus balayani* (Rouba et al. 2024). In contrast, other studies performed in Europe (Spain, France, Italy, Sweden, Switzerland, Norway, Greece) reported lower frequency of HEV RNA in urban sewage, ranging from 3 to 43% (Bisseux et al., 2018; Clemente-Casares et al., 2003, 2009; Kokkinos et al., 2011; La Rosa et al., 2010; Masclaux et al., 2013; Myrmel et al., 2015; Pina et al., 1998). This discrepancy could be due to differences in HEV human epidemiology or in the methods used for viral concentration, nucleic acid extraction and amplification.

In our study, in which the same analytic method was applied, a high frequency of HEV detection in wastewater was observed in Toulouse site, which was expected considering the high frequency of plasma HEV RNA detection in blood donations (incidence rate 2.4/1000), but also in Dunkerque site where the estimation of incidence rate in blood donors was approximately 3 times lower (incidence rate 0.7/1000). Several hypotheses can be proposed. First, the HEV RNA positivity rate in the population served by Dunkerque WWTP during the study period was higher than the positivity rate among blood donors in Dunkerque area. Local heterogeneity of HEV prevalence and incidence have been reported in France (Mansuy et al., 2015, 2016) and in Italy (Spada et al., 2022). Second, potential effects of dilution on ddPCR inhibitors due to the combined sewer system at Dunkerque WWTP could optimize HEV RNA detection. It has been underlined for SARS-CoV-2 that comparing quantitative values between different WWTPs is challenging and that longitudinal measures in wastewater across a large population reflected better accurate trends over time (Parkins et al., 2024). Third, Dunkerque WWTP could contain animal feces. Wastewater samples analyzed in this study (Toulouse and Dunkerque) were tested for porcine adenoviruses and *Prevotella* using methods previously reported (Masclaux et al., 2013; Mieszkin et al., 2009) and found negative for these markers. In addition, cross-reactivity due to the presence of *Roccahepevirus ratti* was excluded because the primers of digital PCR used in this study targeted only *Paslahepevirus balayani* genome.

HEV diversity was analyzed using long-read single-molecule real-time sequencing targeting the *orf2* region. The choice of this region allowed accurate assignments of HEV genotypes and subtypes using reference sequences as recently reported (Hakze-van der Honing et al., 2024). In addition, the long reads and the PacBio CCS mode allowed the characterization of the different haplotypes of each strain and facilitated the identification of HEV strain mixtures. These latter were more frequently identified in wastewater samples (26% at Toulouse site and 37% at Dunkerque site) than in HEV infected individuals (less than 1% based on the National Reference Center data). This information can be used for a better estimation of the theoretical number of infected people in the population connected to a WWTP. Interestingly, HEV diversity observed in wastewater at Toulouse WWTP was similar to HEV diversity in blood donors from Occitanie region or from Toulouse city. The proportion of the predominant subtype (HEV-3c) was identical, as was that observed for HEV-3h, which is a subtype rarely detected. However, it is important to note that the strain 3 h identified in wastewater was slightly genetically distant from the strain 3 h identified in a blood donor living in Toulouse and connected to Toulouse WWTP. In addition, this blood donor was infected in December 2023 after the detection in wastewater (October 2023). A different figure was observed for Dunkerque site. The distribution of HEV-3 subtypes was markedly different between wastewater samples and blood donors from Haut-de-France although subtype 3c remained predominant in both cases. The higher proportion of HEV-3f detected in wastewater samples compared to blood donors could be due to an underestimation of the proportion of HEV-3f in the population connected to the wastewater treatment plant. This latter could not be directly assessed because only one HEV positive blood donor living in Dunkerque was identified (HEV-3c) and the number of blood donors tested was very small at the region scale.

Wastewater-based epidemiology not only involves measuring the concentration of pathogens in raw wastewater downstream but also involves addressing excretion dynamics upstream, accounting for viral decay in sewers or during storage prior to detection, and evaluating actual water use data for general population frequency estimation of positives. There are various models making it possible to establish the proportion of infected people from flow data measured in wastewater, some of which are complex and integrate several parameters. However, an inaccurate determination of these parameters linked to the virus can contribute to the propagation of errors in the calculation of viral excretion upstream in the stools and therefore to a false estimate of infected cases. For example, seasonal change in temperature can alter biological decomposition kinetics, thereby influencing the detectability of downstream pathogens (Hart & Halden, 2020) and introducing more opportunities for false estimates. Additionally, the discrepancy between downstream observations and original inputs could be higher due to increased travel time through the sewers. The variabilities of these parameters must be taken into consideration to guarantee reliable retro-estimation. The idea here was therefore not to introduce a complex model on the basis of parameters whose uncertainties could not be determined but rather to use a simple model and to evaluate from this model the concordance of the kinetics of the proportion of infected people obtained from data in blood and that obtained from data collected in wastewater. It was a linear model which therefore did not take into account several parameters having an impact on the infection, unlike certain more complex models based on flow data measured in wastewater that integrate several parameters influencing the diffusion of viruses in water (decay, transit time, shedding variability, infectivity time, strength of infection) (McMahan et al., 2021; Nourbakhsh et al., 2022). Linear model was therefore necessarily limited in its precision. However it has also been shown in the case of SARS-CoV-2 wastewater epidemiology that simple linear models could have better results than more complex models (Joseph-Duran et al. 2022). Previous studies have shown that there is a good correlation between peak norovirus RNA concentration in wastewater and outbreaks in sampled watersheds, confirming that wastewater screening is a useful approach to monitor norovirus circulation in the community (Kazama et al., 2016). Another recent study found positive correlations between wastewater viral concentration and human test percent positivity for SARS-CoV-2, influenza, and RSV (Lininger et al., 2024). In the case of HEV, the measurement of viral flow rates in wastewater therefore makes it possible to monitor almost in real time the incidence of HEV in the served population. Depending on the time of sampling or the city, a lag of − 1 week to + 3 weeks was observed between the frequencies of positive HEV RNA obtained via wastewater and that observed in blood donors. This variation can be explained by those of the viral load in stools depending on the time of infection, on the duration of HEV shedding in the stools (Kamar et al., 2017) and by differences in sewer systems. This can also be explained by the differences in representativeness of the samples from water and samples from blood donors. Samples from blood donors, even if they were very numerous, could not be fully representative of the general population due to age restrictions for donation (no children and no individuals over 70 years old). Conversely, data from water were probably representative of the general population but they were not collected on an individual scale and the same individual could contribute to the detection of the virus in water over several time points due to extended fecal shedding. HEV RNA positivity frequencies collected weekly from a large population of blood donors can be compared to HEV incidence data for the cities concerned. However, it is important to note that the correlation between the peaks of positive HEV RNA and that in blood donors for Dunkerque was weaker than for Toulouse. It is possible that the blood donor population in Dunkerque was less representative of the general population than that of Toulouse. Since HEV incidence was lower in Dunkerque than in Toulouse, it is also possible that the same infected individuals were detected by the model from one week to the next. HEV RNA positivity frequencies from wastewater flow rates cannot be equated to incidence when a sick individual excretes the virus in the stools over several weeks. It is interesting to note that despite this fact, a simple corrected calculation of HEV RNA positivity frequencies established from wastewater flow rates makes it possible to approximate the incidence in the real population.

A strength of our study is the use of long-read sequencing with a very good accuracy. This allowed us to determine the composition of haplotypes and to identify HEV strain mixtures. This approach based on PacBio technology was validated for HIV and SARS-CoV-2 in spiking experiments and clinical samples (Raymond et al., 2024; Trémeaux et al., 2023; Vellas et al., 2024). The specificity of our PacBio protocol for HEV is 100%. However, the sensitivity compared to amplifying and sequencing shorter ORF2 fragments has not been evaluated. Further studies on HEV comparing long-read and short-read sequencing on wastewater samples are needed. Our study has also several limitations. First, wastewater samples were collected in only two WWTPs. These sites were chosen because available data indicated distinct HEV infection epidemiological pattern with a hyperendemic status of Occitanie region (Izopet et al., 2015; Mansuy et al., 2016). Other French sites of lower endemicity like Brittany should be investigated (Laperche et al., 2024). Second, the study period was only 1 year. This is probably not sufficient to distinguish peaks in HEV RNA concentration due to clusters of asymptomatic infections in the local population from an endemic context of continuous release of HEV RNA in wastewater. Third, the influence of HEV symptomatic acute infections or chronic infections in immunocompromised patients was not analyzed. Fecal shedding of HEV is more important and prolonged in these individuals for whom HEV RNA concentration in blood is higher compared to asymptomatic infected individuals (Lhomme, J. Viral Hepatitis, 2010). However, less than 5% of HEV infections are symptomatic (Abravanel et al., 2025; Kamar et al., 2017).

In summary, the finding that HEV-3 can frequently be detected in untreated wastewater at Toulouse and Dunkerque WWTPs reveals this virus is not only endemic in Occitanie but also in Northern France despite the low number of clinical cases and a lower prevalence of asymptomatic infections compared to Southern France. HEV diversity observed in wastewater agreed with HEV diversity in asymptomatic blood donors living in the same city (Toulouse). Regular HEV monitoring in wastewater could provide a detailed picture of local epidemiology and represents a powerful tool for assessing the effectiveness of One Health programs for mitigating HEV spread globally.

## Data Availability

No datasets were generated or analyzed during the current study.
